# A 6-year review of acute post-streptococcal glomerulonephritis at a public children’s hospital in Cape Town, South Africa

**DOI:** 10.1007/s00467-023-06247-8

**Published:** 2024-01-03

**Authors:** Khadija Abugrain, Mignon I McCulloch, Rudzani Muloiwa, Valerie A Luyckx, Heloise Buys

**Affiliations:** 1 Department of Paediatrics and Child Health, University of Cape Town, Cape Town, South Africa; 2 Division of Paediatric Nephrology, Red Cross War Memorial Children’s Hospital, Klipfontein Road, Cape Town, 7700 South Africa; 3 Renal Division, Brigham and Women’s Hospital, Harvard Medical School, Boston, MA USA; 4 Department of Public and Global Health, Epidemiology, Biostatistics and Prevention Institute, University of Zurich, Zürich, Switzerland; 5 Division of Ambulatory and Emergency Paediatrics, Red Cross War Memorial Children’s Hospital, Cape Town, South Africa

**Keywords:** Acute post-streptococcal glomerulonephritis, Group A streptococci, Haematuria, Children

## Abstract

**Background:**

Acute post-streptococcal glomerulonephritis (APSGN) is the most common cause of acute nephritis in children globally and, in some cases, may be associated with progressive kidney injury and failure, cumulating in the need for long-term dialysis and/or kidney transplantation.

**Methods:**

Our retrospective study describes the occurrence of APSGN among children (< 14 years) admitted to a tertiary children’s hospital in Cape Town, South Africa, from January 2015 to December 2020.

**Results:**

Of 161 children who presented with acute nephritis (haematuria, oedema, oliguria, and hypertension), 100 met the inclusion criteria. Demographic, clinical features, laboratory findings, management, and outcome data were collected. APSGN was defined by the clinical presentation of at least two clinical signs of acute nephritis, and low serum complement 3 (C3) level or evidence of a recent streptococcal infection. Most cases of APSGN were associated with streptococcal skin infections: 55/100 (55%); 10/100 (10%) children presented with hypertensive seizures; C3 levels were low in 86/92 (93.5%) children; 94/94 (100%) children had elevated anti-deoxyribonuclease-B (anti-DNase-B) levels; and 80/94 (85%) also had elevated anti-streptolysin O titre (ASOT) at presentation. Eleven (11%) children had a percutaneous kidney biopsy; 4/11 (36%) showed histological features of post-infectious nephritis, and 7/11(64%) also had crescentic glomerulonephritis with immune complex deposits. Sixty-two (62%) children confirmed recovered, and five (5%) progressed to kidney failure, but 29 presumed recovered as they did not return for follow-up to our institution.

**Conclusions:**

Childhood APSGN remains an important health problem in South Africa (SA) with favourable outcomes in most, apart from those with crescentic glomerulonephritis who progressed to kidney failure.

**Graphical abstract:**

**Figure:**
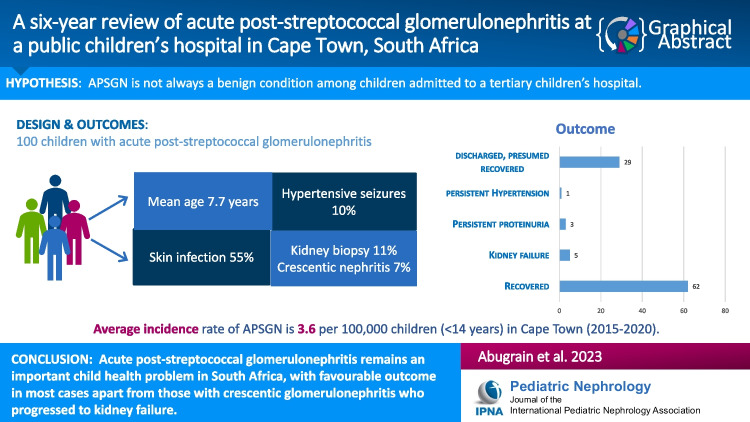
A higher resolution version of the Graphical abstract is available as Supplementary information

**Supplementary Information:**

The online version contains supplementary material available at 10.1007/s00467-023-06247-8.

## Introduction

Glomerular diseases are a major cause of kidney failure in children accounting for 10–15% of kidney failure in children [1]. Post-infectious glomerulonephritis is an immunological response of the kidney following non-kidney infection, most commonly secondary to Group A β-haemolytic streptococcus (GAS) [2], but may also result from other infections, e.g., *Mycoplasma pneumoniae* and *Staphylococcus aureus*. Acute post-streptococcal glomerulonephritis (APSGN), although not a common cause of chronic kidney disease (CKD), is an important cause of paediatric hospital admissions, acute kidney disease (AKD), and parental concern because of the accompanying haematuria and oedema. There has been a decline in the incidence of APSGN in children in high-income countries over the last three decades, with this condition almost completely disappearing in Central Europe [3]. However, this is not the case in other lower socio-economic settings, such as parts of South Africa (SA), where APSGN is still a public health problem, and a frequent cause of paediatric hospital admission and AKD. APSGN in SA has not been studied adequately in recent times and is not currently addressed as an important public health issue. The epidemiology and geographical distribution of streptococcal infection-related glomerulonephritis are not well known across the country and are likely under-reported. Our study aimed to address this knowledge gap.

## Methods

This retrospective descriptive study was conducted from January 2015 to December 2020 at the Red Cross War Memorial Children’s Hospital (RCWMCH), Cape Town, SA. The hospital’s electronic database with recorded International Statistical Classification of Diseases (ICD-10) discharge codes was used to identify all cases of acute nephritic syndrome. Children were included if they presented with at least two signs of acute nephritis syndrome (haematuria, oedema, oliguria, and hypertension), associated with evidence of activation of an alternative pathway complement system (low C3 serum level), or clinical and serological evidence of previous or current streptococcal infection (Table 1), including reported sore throat. Children found to have causes of acute glomerulonephritis other than APSGN were excluded. The Cape Town Census and Population Statistics 2011 database (*Cape Town census and population statistics*) provided the denominator for estimating population-level incidences [4]. The source population (incidence denominator) included the average Cape Town city child (≤ 14 years old) population from the four health districts (Western, Southern, Klipfontein, and Mitchells Plain suburbs) referring to RCWMCH during the study period. Annual incidence per 100,000 calculations was done for the 6-year study period. In a sub-analysis, we looked at the incidence rates per 100,000 children from socio-economically deprived suburbs. The frequency of APSGN was described by geographical area with respect to socio-economic standards and the availability of access to formal housing, clean water, and sanitation. These were seen as potential risk factors contributing to the occurrence of APSGN. Clinical evidence of streptococcal infection was assumed on either a history of recent sore throat or clinical examination showing impetigo or scarring indicating healed recent impetigo. Serological marker levels of streptococcal infection were recorded using anti-streptolysin O titres (ASOT) and/or anti-deoxyribonuclease B antibodies (anti-DNase-B). Generic upper limits of normal values defined by the SA National Health Laboratory System using laser nephelometry technique (Beckman Coulter) were used. For ASOT, the upper limits of normal values are age-specific and are given as follows: < 6 years 100 IU/ml, 6–12 years 250 IU/ml, > 12 years 200 IU/ml. According to local laboratory reference ranges, the normal anti-DNase-B reference range for children is < 75 IU/ml. A pathological diagnosis of APSGN was based on the characteristic pathological features demonstrated by the histological finding of diffuse exudative hypercellular glomerulonephritis, with prominent endocapillary hypercellularity (neutrophils) on light microscopy. On immunofluorescence, the deposition of C3/IgG and IgM in a granular pattern in the mesangium and glomerular capillary wall was seen, and dome-shaped subepithelial deposit “humps” were seen on electron microscopy. Crescentic glomerulonephritis was defined by the presence of ≥ 50% of glomeruli with cellular or fibrous crescents. Continuous data were presented as median and interquartile ranges (IQR) or means and standard deviation (SD), depending on the normality of the data, while proportions of categorical data were presented as percentages. This study was conducted in accordance with the 2013 Declaration of Helsinki and was approved by RCWMCH administration and the University of Cape Town’s Human Research Ethics Committee (HREC) (HREC: 623/2020).

**Table 1:** Operational definitions

Parameter	APSGN definition
Oliguria	Urine volume less than 0.5 mL/kg/h
Clinical oedema	Puffiness of the face, bilateral pitting pedal oedema, and abdominal wall oedema
Microscopic haematuria	Blood in the urine that is detectable by a bedside urine dipstick ≥2 +
Macroscopic haematuria	Frankly blood-stained, visible pink or brown coloured urine owing to the presence of RBC—confirmed by microscopic examination
Proteinuria	• Used normal values of protein excretion as a function of age [5] • Children 1–2 years > 0.04 g protein/mmol creatinine • Children 2–3 years > 0.03 g protein/mmol creatinine • Children 3–5 years > 0.02 g protein/mmol creatinine • Children >5 years > 0.015 g protein/mmol creatinine
Nephrotic range proteinuria	Spot urine protein/creatinine ratio of > 0.2 g/mmol or 3 + of protein on the urine dipstick [6]
Acute kidney injury*	Age-specific normal ranges of enzymatic creatinine were used to diagnose AKI as per the following references [5] • Children < 2 years old 9–32 μmol/L • Children 2 to < 5 years old 18–38 μmol/L • Children 5 to < 12 years old 27–54 μmol/L • Children > 12 years old 40–72 μmol/L
Acute nephritis	A syndrome of AKI, hypertension, haematuria, proteinuria, and fluid overload [7]
Rapidly progressive glomerulonephritis	Glomerular disease (proteinuria, haematuria, and red cell cast) accompanied by rapid loss of kidney function with rising creatinine over days to weeks [7]

*AKI*, acute kidney injury; *RBC*, red blood cells

*GFR was not calculated as routine height measurements were not obtained in the majority of cases; hence, age-specific normal ranges of enzymatic creatinine were used to diagnose AKI

## Results

**Fig. 1:**
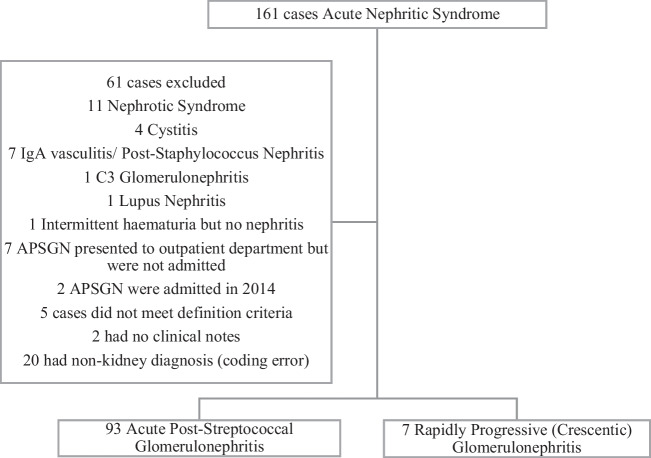
Flow diagram of patient recruitment

During the 6-year period under review, 161 children were identified as having acute nephritic syndrome by ICD-10 coding in the hospital database, 100 met the inclusion criteria and were included, and of these, seven (7%) children had a clinical diagnosis of rapidly progressive glomerulonephritis (RPGN), with crescentic glomerulonephritis due to APSGN in the kidney biopsy (Fig. 1).

**Fig. 2:**
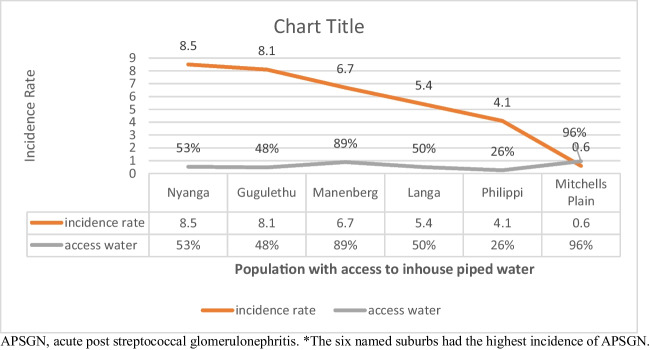
Incidence of APSGN per suburb (the six named suburbs had the highest incidence of APSGN). APSGN, acute post streptococcal glomerulonephritis

Patient characteristics, clinical presentation, diagnoses, and laboratory findings are summarized in Table 2. Of the 55 children with identified skin infections, 10/55 (18%) were treated for impetiginized scabies and 3/55 (5%) for tinea corporis with secondary impetigo. In this study, the population incidence of APSGN in Cape Town was calculated at 3.2 per 100,000 (Table 3). Nevertheless, distribution by communities showed a greater number of cases in communities with a lower socio-economic level and poor access to piped water inside their dwellings; Fig. 2 shows the incidences from six such areas.

**Table 2:** Patient characteristics, clinical presentation, diagnoses, and laboratory findings of the study children

	Total number (*N*)	Clinical presentation	Count (percent)
Age (years)		Mean age at presentation	7.7 (SD 3.03)
Sex		Male to female ratio	1.8:1
Nutrition*	81	Normal weight-for-age Moderate underweight-for-age Severe underweight-for-age	79 (97.5) 2 (2.5) 0 (0)
HIV infection		Screened for HIV infection HIV infected	78 (78) 0 (0)
Clinical presentation with nephritic syndrome	100	Haematuria	99 (99)
		Sub-nephrotic range proteinuria	40 (40)
		Nephrotic-range proteinuria^**#**^	49 (49)
		Oedema	87 (87)
		Oliguria/anuria	31/1 (31/1)
		Hypertension stage 1	7 (7)
		Hypertension stage 2	75 (75)
		Hypertensive encephalopathy	10 (10)
		PRES on brain imaging	8 (8)
Source of streptococcal infection	100	Skin infection	55 (55)
		Throat infection	23 (23)
		Skin and throat infection	10 (10)
		No source identified	12 (12)
Serum C3 level	92	Low C3	86 (93.5)
		Normal C3	6 (6.5)
Antistreptococcal serology	94	Elevated anti-DNase-B	94 (100)
		Elevated ASOT	80 (85)
Diagnosis	100	APSGN	93 (93)
		RPGN crescentic	7 (7)

*C3*, complement 3; *HIV*, human immunodeficiency virus; *AS*, antistreptococcal serology; *APSGN*, acute post-streptococcal glomerulonephritis; *RPGN*, rapidly progressive glomerulonephritis; *PRES*, posterior reversible encephalopathy syndrome

*Nutritional assessment based on WHO weight-for-age *Z*-scores using discharge weights

**#**Six patients had elevated blood cholesterol (>5 mmol/L), low serum albumin (<30 g/L) levels, and nephrotic-range proteinuria on presentation

**Table 3:** Annual incidence of APSGN per 100,000 in children under 14 years of age in Cape Town (2015–2020)

Year	2015	2016	2017	2018	2019	2020	6-Year average
Population size	494,213	501,748	509,097	515,207	519,189	521,457	510,152
Cases per year	11	19	23	19	19	9	16.6
Incidence rate	2.2	3.7	4.5	3.6	3.6	1.7	3.2

*APSGN*, acute poststreptococcal glomerular nephritis

## Seasonal pattern and variation

The seasonal distribution pattern over the 6-year study period showed a higher incidence of APSGN in autumn (March–May) with 35 (35%) cases out of the 100, while the lowest incidence was noted in the spring (September–November) season with 15 (15%) cases. The number of cases in winter and summer was 25 (25%) each. The highest number of cases (23) was noted in the year 2017 and lowest in 2020 (nine cases).

## Management

Fifty-nine (59%) children presented with AKD. Diuretics (furosemide) were administered in 92 (92%) children. Sixty-two (62%) children required amlodipine in conjunction with the diuretic either as a single rescue dose (27) or regular dosage for more than 1 day (35). Eight (8%) patients were admitted to the paediatric intensive care unit, of whom six (6%) were admitted for observation due to recurrent hypertensive seizures, and two (2%) were admitted for cardiorespiratory support due to severe congestive cardiac failure and pulmonary oedema.

Eleven children required a kidney biopsy. The indications for kidney biopsy were either the clinical presentation with RPGN, or nephritic nephrotic presentation (nephritis and nephrotic range proteinuria, hypoalbuminemia, and hypercholesterolemia). All biopsies showed histological changes of diffuse exudative proliferative glomerulonephritis on light microscopy, fibrinogen, and C3 on immunofluorescent staining and dome-shaped subepithelial deposits (humps) on electron microscopy in keeping with post-streptococcal nephritis glomerular damage. Furthermore, seven of 11 (64%) biopsies confirmed cellular or fibrous crescents in more than 50% of the glomeruli in keeping with crescentic glomerulonephritis. Furthermore, all crescentic glomerulonephritis biopsies also showed light, immunofluorescent, and electron microscopy findings confirming APSGN as an underlying pathology. The 10 children who presented with severe nephritis, oliguria, and persistently rising serum creatinine levels despite diuretic use were treated as RPGN. Four of the 10 responded to methylprednisolone (10 mg/kg) pulse therapy, while six needed the addition of cyclophosphamide.

## Outcome

No deaths occurred during the acute presentation and follow-up period. The median length of hospital stay was 5 (IQR 3–6) days and ranged between 1 and 30 days. Twenty-nine of the children had mild clinical presentation and normal kidney function. These children were discharged to another healthcare facility for continuation of care and follow-up evaluation. Hence, these 29 children were presumed recovered. Sixty-two (62%) had resolution of active nephritis, one (1%) had persistent hypertension requiring amlodipine therapy, and three (3%) patients had persistent proteinuria after 6 months (Table 4).These children maintained normal kidney function during follow-up visits but were subsequently lost to follow-up. Outcomes were noted to be unfavourable in children treated for RPGN, as five (5%) progressed to kidney failure. Of these five, three required short-term haemodialysis but did not recover, and long-term intermittent peritoneal dialysis was initiated prior to discharge (two of them had successful kidney transplantation). One patient did not require short-term haemodialysis, yet still progressed to kidney failure and was started on long-term peritoneal dialysis within 6 months of initial presentation. One child defaulted follow-up after discharge and returned in kidney failure 3 years post-discharge. At the 6-week review visit, 69/100 (69%) children were seen. Urine dipstick screening was performed in all children, while three children also had a follow-up of kidney function and complement levels. No child had persistent macroscopic haematuria; 57/69 (83%) had microscopic haematuria, 15/69 (22%) had proteinuria, and 8/69 (12%) had persistent hypertension. Follow-up kidney function and complement levels were normal in the three children in whom these were measured.

**Table 4:** Outcomes of children with APSGN and RPGN

Outcome	APSGN	RPGN	Count (%)
Recovered	62		62 (87.5)
Kidney failure		5	5 (7)
Persistent proteinuria longer than 6 months post episode	2	1	3 (4)
Hypertension on amlodipine, then lost to follow-up	1		1 (1.5)
Discharged (presumed recover)	28	1	29
Grand total	93	7	100

*APSGN*, acute post-streptococcal glomerulonephritis; *RPGN*, rapidly progressive glomerulonephritis

## Discussion

This retrospective analysis of 100 children presenting to RCWMCH with APSGN shows that APSGN is still an important public health problem, is a frequent cause of paediatric hospital admission for AKI in the Western Cape, SA, and is an important contributor to kidney failure in children. All children with RPGN require appropriate follow-up by paediatric kidney services, as some may progress to kidney failure.

The diagnosis of APSGN is usually not difficult when a classical clinical nephritis presentation (haematuria, oedema, oliguria, and hypertension) is associated with serologic evidence of recent streptococcal infection and depressed serum C3 concentration. In this study, serum C3 levels were found to be depressed in 93.5% of children. Five children had both low C3 and low C4 at presentation; however, none of them had clinical or biochemical evidence suggestive of lupus nephritis. All of these five children had a history of either previous or current throat or skin infections with an elevated serum antistreptococcal marker. Rodríguez et al. described the activation of the classical complement pathway (reduced C4 level) in 15–30% of APSGN children [8]. ASOT and anti-DNase-B titre tests are widely available and are the antibodies most frequently elevated in streptococcal upper respiratory and skin infections, respectively [9]. In our cohort, almost two-thirds of children had preceding skin infections, and the anti-DNase-B level was elevated in all of the children where this was measured, suggesting that APSGN in SA is more commonly associated with skin infections than pharyngitis. This pattern of infection is similar to that seen in areas with a temperate Mediterranean climate, such as is the case in Chile [10]. In contrast, other parts of the world, such as Australia and North Africa, have reported APSGN to occur more commonly post-pharyngitis with prevalences of 45% and 80%, respectively [11, 12]. While most children presented with acute nephritis, more than one-third of the children presented with intravascular fluid expansion, pulmonary oedema, congestive cardiac failure, or hypertensive seizures which may suggest late presentation to the healthcare facility.

There is no specific therapy to cure APSGN, and management is mainly based on the supportive treatment of symptoms and complications as required; eradication of the streptococcal infection is also often used, though this is contentious in some countries where antibiotic treatment is only indicated in cases of confirmation of active skin or throat infection [13]. At our institution, a 10-day course of penicillin or, in allergic individuals, erythromycin is standard therapy for streptococcal eradication [14]. The intention for this course of treatment is to prevent the spread of infection to household contacts.

A kidney biopsy is not routinely indicated in the acute setting for the diagnosis of classical APSGN [8]. In our cohort, 11 children required a kidney biopsy. Crescentic glomerulonephritis with immune complex deposits accounted for 64% of APSGN biopsies. This result concurs with what has been described in SA in two separate studies. One study was a 10-year review of native kidney biopsy records at the RCWMCH from 2004 to 2015, and the other was a 7-year review from KwaZulu Natal province between 1981 and 1987, whereby crescentic glomerulonephritis accounted for 5.1% and 5.8% of native kidney biopsies, respectively, with almost 40% and 30% of the crescentic glomerulonephritis being due to post-infectious/streptococcal nephritis [15, 16]. The outcome was noted to be poor in the seven children with crescentic glomerulonephritis, as five of them progressed to kidney failure. This is similar to earlier reports from both high- and low- middle-income countries [15, 17, 18].

Though the incidence of APSGN was found to be high in our study (3.2/100,000), it is still likely to be an underestimate of the true burden of this disease in Cape Town for two reasons. Firstly, this study did not capture subclinical cases as only symptomatic patients presenting for medical services were included. Secondly, as this was a single-centre study, it did not account for cases who may have presented to other health facilities in Cape Town. In SA, the incidence of APSGN in children is not reported across the country. Anecdotal reports from one other South African province suggest a decline in the incidence of APSGN in children in recent years (personal communication Petersen K.). Studies in children with APSGN are scarce in sub-Saharan Africa. However, some data from Nigeria reported an APSGN prevalence of 0.1 per 100,000 children in Sokoto and prevalence rates of 1.3% and 0.8% in Calabar and Benin, respectively [19–21]. Nevertheless, it is more likely that many cases were missed in these studies as access to healthcare is even more challenging than in SA. Furthermore, Chile reported a decline in the average rate of APSGN 6.2 per 100,000 inhabitants for the periods from 1980 to 1983 to 1.7 in the subsequent 10 years 1990–1999 [10]. In our study, the incidence of APSGN was highest in 2017 (4.5 per 100,000 children). We speculate that ongoing severe water restriction and overcrowding in our setting may contribute to the transmission of streptococcal skin infections. South Africa has been facing drought for the last 5 years, with certain parts of the country being classified as a disaster region in 2017. Public health messaging and strict water restrictions imposed by the South African government during the period of critical water shortage led to many people attempting to comply with the restrictions by reducing handwashing, bathing, and resorting to ultrashort shower times. In the Western Cape province in particular, residents were faced with the real threat of a “day 0” outcome for the province running out of water [22]. Further, in our cohort, we noted an increased incidence of APSGN in communities with lower socio-economic status and poor access to piped water inside their dwellings, with incidence rates of 8.5 and 8.1 per 100,000 children in Nyanga and Gugulethu, respectively, as compared to the rest of the drainage area collectively where the incidence rate was 2.7 per 100,000 children. It is possible that some children with APSGN were managed by district hospital specialists, which may be responsible for the relatively low APSGN incidence rate for one of the suburbs included (Philippi).

Interestingly, we noted a decrease in the incidence of APSGN during the year 2020, coinciding with the COVID-19 pandemic. This decreased incidence of APSGN may reflect an effect of the lockdown resulting in reducing the risk of exposure and interpersonal transmission of streptococcal throat and skin infections. In contrast, this may be an underestimation of the true incidences due to the de-escalation and disruption of healthcare services, lack of access to transport during this period, and fear of exposure to COVID-19 at healthcare facilities on the part of the community. In addition, more than one-third of the children from our cohort presented in the autumn (March–May), with fewer cases presenting in spring (September–November). This differs from the findings of a study done in Chile, where the seasonal distribution pattern had a bimodal tendency, with an increase in the autumn (38%) and in spring (30%). The authors did not note any seasonal variation in terms of preceding skin or throat infection [10].

The present study has several limitations which resulted from its retrospective nature. In this study, we made assumptions of post-streptococcal infection in children who presented with either sore throat or impetiginized skin lesions but who did not all have streptococcal serology performed. Other causes of acute post-infectious GN are very rare in our setting. In the public health sector of South Africa, throat and skin infections are rarely confirmed by laboratory diagnosis with most being made clinically. This practice does raise challenges in estimating and assessing the burden of streptococcal infections in relation to post-infectious glomerulonephritis. In addition, this is a report from a single centre and the follow-up of affected children was inadequate, with most patients absconding from follow-up after their first visit. Yet, our study has several strengths. This is a large cohort of children who attended a public children’s hospital, and we have been able to show that APSGN is still an important public health condition and that this may be related to poverty, overcrowding, infection, and infestations such as scabies and lack of regular access to piped water.

Based on our observations, where 10% of children presented as RPGN requiring a kidney biopsy and subsequent therapy with immune modulators, we recommend that all children presenting acutely with oedema or haematuria should have their blood pressure and serum creatinine levels measured. If the serum creatinine is normal, then RPGN is unlikely, a favourable outcome is more likely, and the child can be followed as an outpatient. A raised serum creatinine may be more ominous, however, and we recommend that these children be referred for evaluation at a tertiary children’s centre. We also recommend that in addition to ASOT titres which are routinely measured in these cases, anti-DNase-B levels should also be measured as in our cohort, as skin infections appear more common in lower resource settings. We further recommend that all children be followed up at least once at 6 weeks post-episode, where their blood pressure, urine dipstick, serum complement, serum creatinine, and general wellness can be measured. Given the high loss to follow-up in our cohort, supplying families with information leaflets for education and stressing the importance of follow-up at discharge is important. One child developed kidney failure, which may have been delayed or prevented had the child been followed up. Due to the retrospective nature of the study, we noted that the follow-up of about one-third of affected children was inadequate. This is an important limitation and is somewhat difficult to address practically as every journey to a hospital costs the family money that is often meant for subsistence. One possible way to ensure medical review closer to home could involve collaboration with family medicine practitioners who are positioned in the community closer to where the children of this study reside; they could then select children who require specialist assessment.

## Conclusion

APSGN during childhood continues to be an important health problem in SA, more commonly following skin infection than throat infection. Despite a complicated course in some children, the outcome is favourable in most children, apart from those with crescentic glomerulonephritis who mostly progressed to kidney failure. We recommend more attention be paid to the post-discharge follow-up and suggest a leaflet handed to the patient’s caregiver to state the importance of adherence to follow-up even in the absence of haematuria.

### Supplementary Information

Below is the link to the electronic supplementary material.

Graphical abstract (PPTX 54.1 KB)

Supplementary file1 (DOC 91.0 KB)

## Data Availability

The datasets generated during and/or analysed during the current study are available from the corresponding author on reasonable request.

## Declarations

We understand that the corresponding author is the sole contact for the editorial process (including Editorial Manager and direct communications with the office). He/she is responsible for communicating with the other authors about progress, submissions of revisions, and final approval of proofs.

## References

[1] Vinen CS, Oliveira DBG (2003). Acute glomerulonephritis. Postgrad Med J.

[2] Bullen A, Shah MM (2018). De Novo postinfectious glomerulonephritis secondary to nephritogenic streptococci as the cause of transplant acute kidney injury: a case report and review of the literature. Case Rep Transplant.

[3] Rodriguez-Iturbe B, Musser JM (2008). The current state of poststreptococcal glomerulonephritis. J Am Soc Nephrol.

[4] City of Cape Town (2017) Cape Town census and population statistics. https://www.capetown.gov.za-statistics-and-research/cape-town-census. Accessed 16 October 2021

[5] Van der Watt G, Omar F, Brink A, McCulloch M, Avner ED, Harmon WE, Niaudet P, Yoshikawa N, Emma F, Goldstein SL (2016). Laboratory investigation of the child with suspected renal disease. Paediatric nephrology.

[6] Trautmann A, Vivarelli M, Samuel S, Gipson D, Sinha A, Schaefer F (2020). IPNA clinical practice recommendations for the diagnosis and management of children with steroid-resistant nephrotic syndrome. Pediatr Nephrol.

[7] Rees L, Bockenhauer D, Webb NJ, Punaro MG (2019). Paediatric nephrology.

[8] Rodriguez-Iturbe B, Najafian B, Silva A, Alpers CE (2016). Acute postinfectious glomerulonephritis in children. Pediatr Nephrol.

[9] Kanjanabuch T, Kittikowit W, Eiam-Ong S (2009). An update on acute postinfectious glomerulonephritis worldwide. Nat Rev Nephrol.

[10] Berríos X, Lagomarsino E, Solar E, Sandoval G, Guzmán B, Riedel I (2004). Post-streptococcal acute glomerulonephritis in Chile—20 years of experience. Pediatr Nephrol.

[11] Elzouki A, Amin F, Jaiswal O (1983). Prevalence and pattern of renal disease in eastern Libya. Arch Dis Child.

[12] Blyth CC, Robertson PW, Rosenberg AR (2007). Post-streptococcal glomerulonephritis in Sydney: a 16-year retrospective review. J Paediatr Child Health.

[13] Roy S, Stapleton FB (1990). Changing perspectives in children hospitalized with poststreptococcal acute glomerulonephritis. Pediatr Nephrol.

[14] Ståhlgren GS, Tyrstrup M, Edlund C, Giske CG, Mölstad S, Norman C (2019). Penicillin V four times daily for five days versus three times daily for 10 days in patients with pharyngotonsillitis caused by group A streptococci: randomised controlled, open label, non-inferiority study. BMJ.

[15] Mwaba C (2017). A ten-year retrospective study of the aetiology and outcome of crescentic glomerulonephritis in children presenting to the Red Cross Children's Hospital, Cape Town, South Africa (MA thesis).

[16] Parag KB, Naran AD, Seedat YK, Nathoo BC, Naiker IP, Naicker S (1988). Profile of crescentic glomerulonephritis in Natal - a clinicopathological assessment. Q J Med.

[17] Tapaneya-Olarn W, Tapaneya-Olarn C, Boonpucknavig V, Boonpucknavig S (1992). Rapidly progressive glomerulonephritis in Thai children. J Med Assoc Thai.

[18] Dewan D, Gulati S, Sharma RK, Prasad N, Jain M, Gupta A (2008). Clinical spectrum and outcome of crescentic glomerulonephritis in children in developing countries. Pediatr Nephrol.

[19] Jiya FB, Ibitoye PK, Jiya NM, Abba MH (2021). Acute post streptococcal glomerulonephritis among children from Sokoto, North-Western Nigeria. Asian J Pediatr Res.

[20] Ibadin O, Abiodun P (2003). Childhood acute glomerulonephritis in Benin City. Niger J Paediatr.

[21] Etuk I, Anah M, Eyong M (2009). Epidemiology and clinical features of acute glomerulonephritis in Calabar, Nigeria. Niger J Physiol Sci.

[22] World Economic Forum (2019) Cape Town almost ran out of water. Here's how it averted the crisis. https://www.weforum.org/agenda/2019/08/cape-town-was-90-days-away-from-running-out-of-water-heres-how-it-averted-the-crisis/. Accessed 15 August 2021

