# Supplementary material for: A 6-year review of acute post-streptococcal glomerulonephritis at a public children’s hospital in Cape Town, South Africa
Source: Pediatr Nephrol. 2024 Jan 3;39(6):1809–16. doi: 10.1007/s00467-023-06247-8 (PMC11026274; doi:10.1007/s00467-023-06247-8)
Supplement: Supplementary file 1 — Graphical abstract (PPTX 54.1 KB) [file 467_2023_6247_MOESM1_ESM.pptx]

## Slide 1
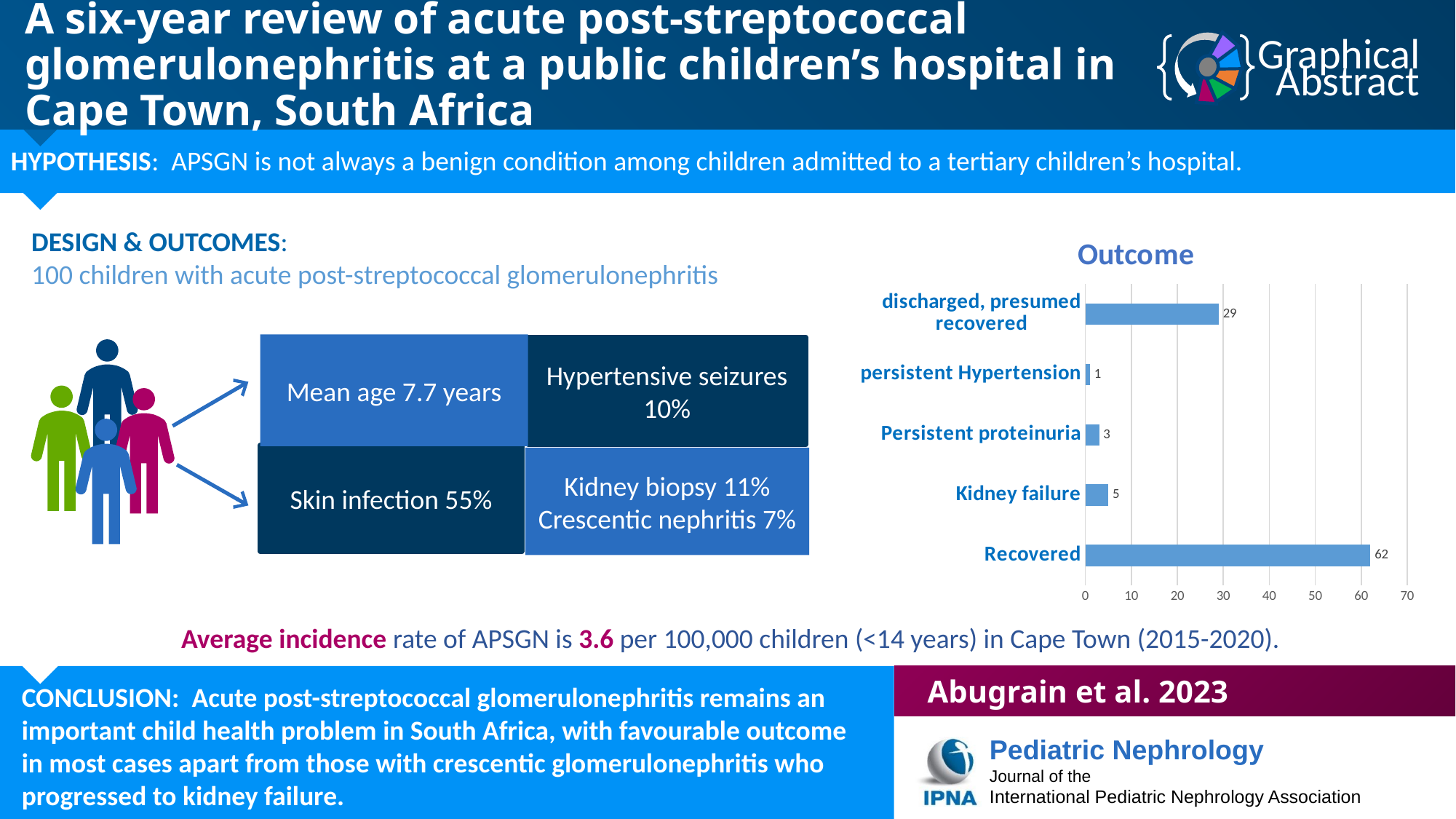

A six-year review of acute post-streptococcal glomerulonephritis at a public children’s hospital in Cape Town, South Africa
HYPOTHESIS: APSGN is not always a benign condition among children admitted to a tertiary children’s hospital.
DESIGN & OUTCOMES:
100 children with acute post-streptococcal glomerulonephritis
Average incidence rate of APSGN is 3.6 per 100,000 children (<14 years) in Cape Town (2015-2020).
### Chart: Outcome
| Category | Column2 |
|---|---|
| Recovered | 62.0 |
| Kidney failure | 5.0 |
| Persistent proteinuria | 3.0 |
| persistent Hypertension | 1.0 |
| discharged, presumed recovered | 29.0 |Mean age 7.7 years
Hypertensive seizures 10%
Skin infection 55%
Kidney biopsy 11%
Crescentic nephritis 7%
Abugrain et al. 2023
CONCLUSION: Acute post-streptococcal glomerulonephritis remains an important child health problem in South Africa, with favourable outcome in most cases apart from those with crescentic glomerulonephritis who progressed to kidney failure.
